# Comparative genomics-based investigation of resequencing targets in *Vibrio fischeri*: Focus on point miscalls and artefactual expansions

**DOI:** 10.1186/1471-2164-9-138

**Published:** 2008-03-25

**Authors:** Mark J Mandel, Eric V Stabb, Edward G Ruby

**Affiliations:** 1 Department of Medical Microbiology and Immunology, University of Wisconsin School of Medicine and Public Health, 1550 Linden Drive, Madison WI 53706-1521, USA; 2 Department of Microbiology, University of Georgia, 828 Biological Sciences, Athens, GA 30602-2605, USA

## Abstract

**Background:**

Sequence closure often represents the end-point of a genome project, without a system in place for subsequent improvement and refinement. Building on the genome project of *Vibrio fischeri *ES114, we used a comparative approach to identify and investigate genes that had a high likelihood of sequence error.

**Results:**

Comparison of the *V. fischeri *ES114 genome with that of conspecific strain MJ11 identified 82 target loci in ES114 as containing likely errors, and thus of high-priority for resequencing. Analysis of the targets identified 75 loci in which an error had occurred, resulting in the correction of 10,457 base pairs to generate the new ES114 genomic sequence. A majority of the inaccurate loci involved frameshift errors, correction of which fused adjacent ORFs. Although insertions/deletions are thought to be rare in microbial genome assemblies, fourteen of the loci contained extraneous sequence of over 300 bp, likely due to imperfect contig ends that were misassembled in tandem rather than as overlapping segments. Additionally we updated the entire genome annotation with 113 new features including previously uncalled protein-coding genes, regulatory RNA genes and operon leader peptides, and we analyzed the transcriptional apparatus encoded by ES114.

**Conclusion:**

We demonstrate that errors in microbial genome sequences, thought to largely be confined to point mutations, may also consist of other prevalent large-scale rearrangements such as insertions. Ongoing genome quality control and annotation programs are necessary to accompany technological advancements in data generation. These updates further advance *V. fischeri *as an important model for understanding intercellular communication and colonization of animal tissue.

## Background

In the thirteen years since the announcement of the first complete organism genome [1], there has been a rapid accumulation of sequence data from complete and draft genomes. The number of complete or almost-complete projects is in the range of 3,000 [2], but this number is a "moving target," and improvements in sequencing technologies over the past decade ensure continued rapid expansion in the number and diversity of organisms that are analyzed by complete genome sequencing.

Despite these significant advances in data acquisition, there have not been commensurate improvements in data-quality assessment and refinement during this period. Individual miscalled bases are assumed to be present in practically all completed genome sequences, and their frequency has been suggested to be between 1–100 errors per 100 kb [3] and has been measured in some instances to be at most 1 error per 88 kb [1,4]. Errors in microbial genomes are believed to be generally restricted to point miscalls, with large-scale rearrangements rarely occurring [3]. To identify and correct errors, recent studies have utilized microarray-based detection, in which errors in a subject genome are identified by comparison to a reference genome which served as the basis for array construction. For example, this method has been employed successfully in *Escherichia coli *[5] and *Bacillus anthracis *[6]. However, these analyses are unidirectional: "errors" are defined as sequence distinct from that of the reference genome, and therefore errors in the reference genome cannot be detected.

As small nucleotide changes in a genome model often manifest as large protein errors – for instance, due to introduction of frameshift and nonsense errors – multiple approaches have capitalized on this protein signal to detect DNA errors in complete genomes [7-10]. By comparing protein-coding sequences in a subject strain to those in a closely-related strain or to closely-related proteins in molecular databases, one can identify those that are potentially truncated inappropriately in the subject strain and target those regions for resequencing. Targeted resequencing has been applied successfully in *B. subtilis *[10] and *Mycobacterium smegmatis *[11], and in both cases the errors were restricted to changes in 1–2 nucleotides. Importantly, Perrodou et al. [8] generalized this method *in silico *to make it available to any subject organism of interest. Targeted resequencing is efficient and available to a wide range of investigators because: (i) the initial steps are completed *in silico *prior to proceeding to the wet laboratory; and (ii) when a closely-related strain is available targeted resequencing provides an efficient means to identify discrepancies that alter coding sequence predictions.

In this study, we focus on the genome of the luminous Gram-negative bacterium *Vibrio fischeri *ES114. *V. fischeri *forms symbiotic associations with squid and fish, and the association between *V. fischeri *and the Hawaiian bobtail squid *Euprymna scolopes *represents one of the most powerful natural models for the study of mutualistic animal-microbe relationships. Specific strains of symbiotic *V. fischeri *colonize a dedicated "light organ" in the squid host, multiply to high density, and exhibit luminescence in a density-dependent manner [12,13]. The light produced by the bacteria is believed to aid the squid host by providing protection from predators: the shadow revealed from the nocturnal-foraging squid in moonlight is camouflaged by the downward-welling light of the host-associated *V. fischeri *[14]. In return, the bacterium benefits from a protected, nutrient-rich environment. This was the first system in which it was shown that a specific symbiont directs normal animal development [15], and now represents an emerging model for cross-kingdom genomics-based studies.

The genomic potential for this system is based on a strong history of molecular inquiry on both the symbiont and host sides of the interaction. First, the complete genome sequence of squid symbiont *V. fischeri *ES114 has been published and studied, and the sequence revealed novel insights into pilin gene diversity and the distribution of toxin genes in beneficial bacteria [16]. Second, based on the genome sequence a number of global studies have been initiated; the first sets to be published yield novel results about how chemical communication among *V. fischeri *strains regulates bacterial behavior [17,18] and how two-component signal transduction affects host-interaction [19,20]. Third, an EST library of the squid host [21] has provided novel insight into cephalopod genetic capabilities and widely conserved signaling pathways such as the NF-κB pathway [22]. Fourth, the phenomenon we now call quorum sensing – autoinduced density-dependent cell-cell communication – was first described in *V. fischeri *[23], and a number of evolutionary and modeling studies of this process have focused on the well-characterized systems in *V. fischeri*. Fifth, by having access to the natural host – a rarity among systems in which high-throughput genetic and genomics approaches are applicable – we can exploit the high information content in the coevolved squid-*Vibrio *relationship to learn how closely-related pathogenic marine microbes interact with natural hosts that have yet to be identified. Sixth, the draft genome of a second strain of *V. fischeri*, the fish symbiont MJ11, is being completed and will provide a strong platform for applying comparative genomic approaches to the study of host-specificity.

While undertaking such a comparative study among *V. fischeri *strains, we detected a high incidence of suspected genomic anomalies in the published sequence of *V. fischeri *ES114. We resequenced these suspect regions and identified 91% of these loci to be in error. Notably, in fourteen of the cases we detected misincorporation of extraneous sequence in the published assembly, leading to the appearance of duplicated DNA where none existed. In five other cases, the sequence in the suspect region was correct in the published sequence and the resulting gene product would be predicted to be nonfunctional; we therefore designated these features as pseudogenes in ES114. In addition to correcting these features, we completed a full genomic update of ES114 gene annotations, and incorporated the addition of 113 genes that were previously unannotated into release 2.0 of the ES114 annotation. Together these updates advance *V. fischeri *as a platform for functional and comparative genomic studies, and demonstrate how a targeted set of approaches may yield high impact on genomic quality improvement.

## Results

### Identification of suspect genomic regions

We obtained the draft genome sequence of *V. fischeri *strain MJ11 and, as part of our initial analysis, we conducted a number of reciprocal BLAST analyses to compare its predicted proteome with that of the completely sequenced conspecific strain ES114 [16]. We used BLASTP [24] to identify orthologs between the two strains, using a modified reciprocal best-hit approach as outlined in the Methods. A surprising outcome from this analysis was the occurrence of over seventy protein-coding genes in MJ11 with reciprocal best-hits to two neighboring genes in ES114. At the time that we were performing this analysis, a handful of cases were being identified empirically in which neighboring genes in ES114 were actually one gene, and that the appearance of two genes resulted from frameshift or nonsense errors in the original sequence data. Examples that were identified independent of this work include *ptsI *[25], *fnr *(J.L. Bose and E.V.S., unpublished data), and *acs *(S.V. Studer & E.G.R., unpublished data).

Analysis of the suspect regions supported the hypothesis that there were a large number of loci in ES114 in which sequencing errors had led to the miscalling of one gene as multiple ORFs. In support of this hypothesis, we identified a number of genes that are essential in *Escherichia coli *and other bacteria, but that were split in version 1.0 of the ES114 sequence. These included *dnaG*, *ftsQ*, *mukB*, *nusG*, *rplC*, *rplN*, *rplO*, *rpoB*, *rpoC*, *thrS*, and *tilS *[26,27], and the conditionally-essential *rpoH *[28]. Second, we identified eleven ambiguous bases (i.e., "N" listed in the nucleotide sequence) that had been called in the original sequence, and the incidence of these bases correlated with the presence of suspect ORFs.

In addition to suspected frameshifts and substitutions, we also identified fourteen regions in which it appeared that extraneous sequence had been incorporated that was highly similar to neighboring sequence, with the size of the duplicated/extraneous region ranging from 318 bp to 1264 bp. In one case, pre-genomic sequencing of a suspect region did not identify any repeated sequence [29]. Therefore, we hypothesized that these regions represented assembly errors in which the same stretch of DNA was mistakenly incorporated twice into the genome's sequence. These regions typically contained a few unique base pairs at either end – likely due to low-coverage sequencing – that led to the misincorporation, but were otherwise essentially a direct repeat of DNA that had the effect of introducing extra and/or truncated ORFs.

A list of the loci targeted for resequencing was assembled and each was assigned a "target number"; that number is used consistently in tables and figures so that the primer sequences used to analyze the data may be correlated with the resulting sequence and analysis.

In addition to the BLASTP-based identification of potential errors, we undertook a full-length visual comparison of the chromosomes of *V. fischeri *ES114 and MJ11. Given the prevalence of errors detected by identifying adjacent ORFs that likely represented a single ORF, we hypothesized that there were probably other cases of errors that would not have manifest themselves in this way. Examples of other suspected errors that warranted investigation included situations in which one of the fragmented ORFs was too small to be detected as an ortholog candidate by the BLASTP filters, or in which the second fragment did not lead to a predicted open reading frame. Using the program Mauve [30], we analyzed ORFs along the length of the chromosomes, identifying candidates that had suspect 5' or 3' ends. In some cases, these appeared to result solely from annotation differences, in which identical sequences had predicted translational start sites (5' boundaries) that were called at distinct points in the two annotations. In other cases, sequence differences underlay the unique ORF boundaries, and we targeted those for our analysis. Furthermore, there were three cases in which putative extraneous sequence was visually identified in intergenic sequence, which could not have been detected by BLASTP analysis in the absence of annotated ORFs (target nos. 130, 172, 178). These cases were added to the list of targeted loci. Finally, any remaining ambiguous bases in the sequence were targeted for resequencing.

### Sequence clarification

We examined a total of 82 targets for resequencing. Our general approach involved amplifying across the target, and then sequencing the amplified product with the PCR primers. In cases where we were clarifying the sequence following a large detected "deletion" (missing sequence from what is predicted from the published sequence), we amplified a larger product and sequenced from a set of sequencing primers across both strands. For the oligonucleotide primers used for PCR and sequencing see Additional file 1. With one exception, all of the primer pairs amplified products in which there was a clear, predominant band, and thus served as satisfactory templates for sequencing. The primer pair that failed to amplify (target no. 180) included a primer that was in a region that does not exist in the true ES114 sequence, as clarified by our analysis of target no. 182. Therefore the absence of a band in this case supports the deletion that emerged from target no. 182.

**Figure 1:**
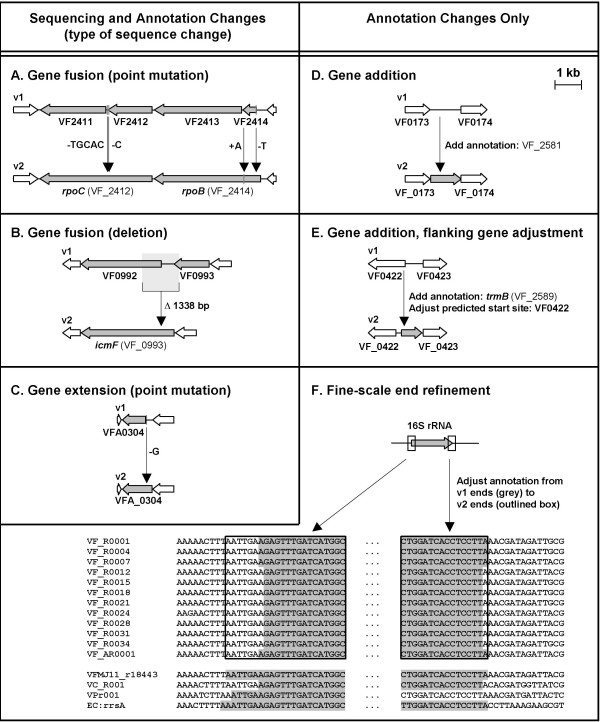
**Types of genomic changes described**. Examples of the types of chromosomal corrections (A-C) and annotation corrections (D-F) described throughout the paper. The case in (B) shows the artefactual expansions that were removed in this analysis. v1 refers to the previously published version 1.0 release, and v2 refers to the version 2.0 release reported here.

Seventy-five of the 82 sets (91%) of resequencing targets examined were found to be in error in the published ES114 sequence. The errors, subsequent changes, new locus tags, and new annotations, are listed in Table 1. Conceptual diagrams of representative sequencing and other annotation changes discussed during this report are illustrated in Figure 1. Note that with this update, the locus tag format has been modified to the new NCBI format for locus tags (underscore following the "VF" prefix, which denotes *V. fischeri *ES114). As a convention, in cases of gene fusion, the locus tag of the 5'-proximal (N-terminal-encoding) fragment retained its locus tag identifier, while the identifier(s) for the remaining gene fragment(s) were deaccessioned.

**Table 1:** *V. fischeri *ES114 loci modified due to sequence changes.

**Locus tag**	**Gene**	**Description**	**Correction**	**s/m**	**Effect on ORFs**	**Locus tag deaccessioned**	**Target**
VF_0040	*yidZ*	transcriptional regulator, LysR family	fs	s	fusion	VF0039	101
VF_0044	*rmuC*	predicted recombination limiting protein	fs	s	fusion	VF0045	102
VF_0056	*rhlB*	ATP-dependent RNA helicase	fs	s	fusion	VF0055	103
VF_0093	*add*	adenosine deaminase	dl	m	fusion	VF0092	104
VF_0124	*slmA*	division inhibitor	fs	s	fusion	VF0123	105
VF_0157	*wbfB*	WbfB protein	fs, ms, n	m	fusion	VF0156	106
VF_0160	*wbfD*	WbfD protein	fs	s	fusion	VF0159	107
VF_0214	*prkB*	phosphoribulokinase	fs	s	fusion	VF0213	109
VF_0220	*kefB*	potassium:proton antiporter	fs, ns	m	fusion	VF0221	110
VF_0235	*rplC*	50S ribosomal subunit protein L3	fs	s	fusion	VF0236	111
VF_0246	*rplN*	50S ribosomal subunit protein L14	fs	s	fusion	VF0247	112
VF_0256	*rplO*	50S ribosomal subunit protein L15	fs	s	3' extension		168
VF_0281	*yjjP*	predicted inner membrane protein	fs	s	fusion	VF0282	113
VF_0300		putative salt-induced outer membrane protein	fs	s	fusion	VF0299	114
VF_0397	*yrbC*	predicted ABC-type organic solvent transporter	fs	s	fusion	VF0398	116
VF_0418	*dgkA*	diacylglycerol kinase	fs	m	3' extension		169
VF_0420	*mltC*	membrane-bound lytic murein transglycosylase C	fs, ms	m	fusion	VF0419	117
VF_0481	*glmM*	phosphoglucosamine mutase	fs	m	fusion	VF0482	118
VF_0651		amino-acid abc transporter binding protein	fs	s	3' extension		170
VF_0657		succinylglutamate desuccinylase/aspartoacylase family protein	n	s	ambiguous residue clarified		179
VF_0729	*nqrE*	sodium-translocating NADH:quinone oxidoreductase, subunit E	fs	s	fusion	VF0730	119
VF_0762	*ychF*	predicted GTP-binding protein	fs, ms	m	fusion	VF0761	120
VF_0960	*tolA*	membrane anchored protein in TolA-TolQ-TolR complex	dl	m	fusion	VF0961	171
VF_0993	*icmF*	secretion protein IcmF	dl	m	fusion	VF0992	182
VF_1031	*trpG*	anthranilate phosphoribosyltransferase	fs	s	fusion	VF1030	122
VF_1214	*thrS*	threonyl-tRNA synthetase	fs	s	fusion	VF1215	123
VF_1304		copper-exporting ATPase	fs	s	fusion	VF1305	125
VF_1308	*fnr*	transcriptional regulatory protein Fnr, global regulator of anaerobic growth	ms, ns	m	fusion	VF1309	183
VF_1358	*fdnI*	formate dehydrogenase N, gamma subunit	fs	m	fusion	VF1357	126
VF_1515		GGDEF domain protein	fs	s	3' extension		185
VF_1669	*menB*	dihydroxynaphthoic acid synthetase	fs	s	fusion	VF1668	127
VF_1771	*prkA*	serine kinase PrkA	dl	m	fusion	VF1772	128
VF_2633		lipoprotein, putative	dl	m	none		172
VF_1828		C-terminal CheW domain, putative chemotaxis coupling protein	fs	s	fusion, 3' extension	VF1827	129
None		Intergenic: VF_1856 – VF_1858	dl	m	deletion	VF1857	130
VF_1895	*ptsI*	PEP-protein phosphotransferase of PTS system (enzyme I)	fs	s	fusion	VF1896	184
VF_1932	*fadE*	acyl coenzyme A dehydrogenase	fs	s	fusion	VF1933	131
VF_1938		hydroxyacylglutathione hydrolase	ms, n	m	amino acid substitutions		121
VF_1945	*tilS*	tRNA(Ile)-lysidine synthetase	dl	m	fusion	VF1944	132
VF_2049	*malZ*	maltodextrin glucosidase	fs, ns	m	fusion	VF2050	133
VF_2078	*mazG*	nucleoside triphosphate pyrophosphohydrolase	fs	s	fusion	VF2077	134
VF_2152	*amtB*	ammonium transporter	fs	s	3' extension		173
VF_2166	*pcnB*	poly(A) polymerase I	fs, ms, ns	m	fusion	VF2167	135
VF_2181	*aceE*	pyruvate dehydrogenase, decarboxylase component E1, thiamin-binding	dl	m	fusion	VF2180	136
VF_2199	*ftsQ*	cell division protein FtsQ	fs	m	fusion	VF2198	137
VF_2220		ubiquinol-cytochrome c reductase iron-sulfur subunit	fs	s	fusion	VF2219	138
VF_2252	*dnaG*	DNA primase	fs, ms, ns	m	fusion	VF2253	139
VF_2347	*cysE*	serine acetyltransferase	fs, ms	m	fusion	VF2346	140
VF_2366	*znuA2*	high-affinity zinc uptake system protein ZnuA2	fs	s	fusion	VF2365	141
VF_2370	*yeiR*	predicted enzyme	fs	s	fusion	VF2371	142
VF_2377		hypothetical protein	dl	m	fusion	VF2378	143
VF_2383	*acs*	acetyl-CoA synthetase	fs	m	fusion	VF2384	144
VF_2389	*dusB*	tRNA-dihydrouridine synthase B	fs, ms	m	fusion	VF2390	145
VF_2412	*rpoC*	RNA polymerase, beta prime subunit	fs	m	fusion	VF2411	146
VF_2414	*rpoB*	RNA polymerase, beta subunit	fs	m	fusion, 5' extension	VF2413	147–148
VF_2418	*rplA*	50S ribosomal subunit protein L1	fs	m	fusion	VF2417	149
VF_2421	*nusG*	transcription termination factor NusG	fs	m	fusion	VF2420	150
VF_2450	*rpoH*	RNA polymerase, sigma-32 (sigma-H) factor	fs, ms, n	m	fusion	VF2449	151
VF_2463	*nudE*	ADP-ribose diphosphatase	fs	s	5' extension		174
VF_2528	*ilvC*	ketol-acid reductoisomerase, NAD(P)-binding	dl	m	fusion	VF2526, VF2527	152
VF_A0046		acriflavin resistance plasma membrane protein	fs	m	fusion	VFA0047	153
VF_A0244		GGDEF/EAL domains protein	fs, dl	m	fusion	VFA0242, VFA0243	154–155
VF_A0251	*fdhF*	formate dehydrogenase-H	fs	m	fusion	VFA0252	156
VF_A0304		hypothetical protein	fs	s	5' extension		176
VF_A0338		putative glucosyl hydrolase precursor	fs	m	fusion	VFA0337	158
VF_A0353	*galT*	galactose-1-phosphate uridylyltransferase	fs	m	fusion	VFA0354	159
VF_A0432	*mukB*	fused chromosome partitioning protein: predicted nucleotide hydrolase	fs, ms	m	fusion	VFA0433	160
VF_A0460	*mfd*	transcription-repair coupling factor	fs	s	fusion	VFA0459	161
None		Intergenic: VF_A0655-VF_A0666	fs, ms, n	m			178
VF_A0832	*putA*	proline dehydrogenase	dl	m	fusion	VFA0831	162
VF_A0856		hypothetical protein	dl	m	fusion	VFA0855	163
VF_A1008		hypothetical protein	fs, ms	m	fusion	VFA1009	165
VF_A1152	*acrA*	multidrug efflux system	fs	m	fusion	VFA1151, VFA1150	166
VF_A1156		ATP-dependent DEXH-box helicase	dl	m	fusion	VFA1157	167

Correction types: dl, large deletion; fs, frameshift; ms, missense; ns, nonsense; n, ambiguous nucleotide. s/m indicates whether (s)ingle or (m)ultiple nucleotides were affected by the sequence change.

**Figure 2:**
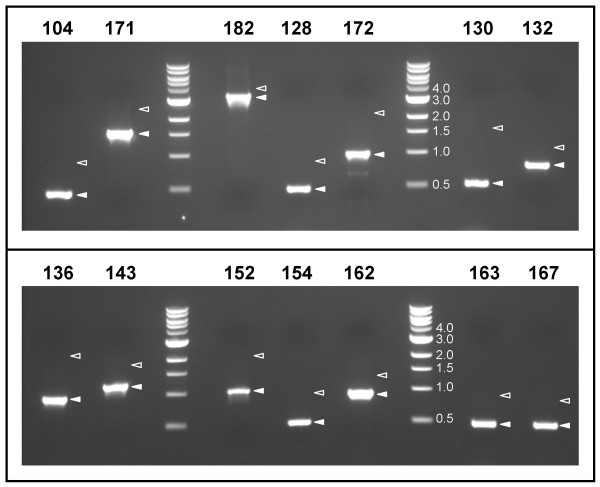
**Evidence of expansions at multiple chromosomal sites**. The fourteen resequencing targets examined had extraneous sequence in the published version. In each case, correction of the error required large deletions (over 300 bp). For each of the targets examined, the closed arrowhead indicates the band observed upon amplification with the PCR primers listed, whereas the open arrowhead indicates the size of the product expected by the sequence in the published version 1.0. Marker sizes are indicated in kb.

It is thought that the creation of false large-scale genomic rearrangements such as insertions rarely occurs in microbial genome projects [3,11]; however, we confirmed the presence of all fourteen predicted insertions by amplifying from the respective unique flanking regions, and demonstrating that the bands obtained are inconsistent with the previous sequence model (Figure 2). In each case, the bands observed were smaller than predicted, and the sequence obtained led to the precise deletion of the extraneous repeated DNA in the new model.

Most of the resulting changes led to the fusion of two – or in some cases three – neighboring ORFs, and/or the extension of ORFs at the 5' or 3' end (Figure 1A–C; Table 1). In one case (target no. 178), the deletion affected only an intergenic region that contains no annotated features. In another case (target no. 172) the deletion identified by visual analysis in Mauve affected what was believed to be a 1498-bp intergenic region between *rluE *and VF_1777. The corrected sequence revealed this region to be only 368 bp in length, but also that it contains a predicted lipoprotein conserved in *V. fischeri *MJ11. The new release reflects the sequence deletion, as well as the added annotation for this gene (VF_2633).

The resulting sequence corrections led us to propose a number of protein annotations that were consistent with our predictions. Based on the corrected sequence, many conserved genes now more closely resemble their orthologs in other species. In other cases, the domain structure of even poorly characterized proteins supported the accuracy of the corrections. For example, target no. 185 extended the 3' end of VF_1515 by correcting a frameshift mutation. Analysis of protein domains by conserved domain search (CDD; [31]) identified an incomplete GGDEF (diguanylate cyclase) domain in the protein's C-terminus, and correction of the frameshift led to inclusion of the entire domain.

### Pseudogenes and degeneration in *umuC*

In some of the cases we confirmed the published ES114 sequence to be correct, and that the ORF boundaries (5' or 3' end, or the presence of two genes instead of one) were correct in ES114 version 1.0. Table 2 lists those five cases that we can now more confidently assume to be pseudogenes in ES114 because they appear to be nonfunctional given their predicted amino acid sequence. In each case, the indicated defect is predicted to interrupt a significant portion of the coding sequence required for function in well-characterized homologs. The N-acetylglucosaminyltransferase VF_A0466 has two (apparently functional) paralogs in the genome, and ES114 is capable of utilizing N-acetylglucosamine as a sole N+C source (data not shown): therefore, the appearance of a pseudogene at this location does not have obvious functional consequences for the cell.

**Table 2:** Pseudogenes described in ES114 version 2.0.

**Locus tag**	**Previous**	**Homolog**	**Defect in *V. fischeri *ES114 versus MJ11**	**Target**
VF_0198	VF0198, VF0199	*ugd*, UDP-glucose 6-dehydrogenase, capsule biosynthetic gene	+1 frameshift	108
VF_1268	VF1267, VF1268	*umuC*, DNA polymerase V subunit	amber nonsense codon and 5 bp repeat expansion	124
VF_A0141	VFA0141	putative transporter, NadC family protein	-1 frameshift	175
VF_A0270	VFA0270, VFA0271	transcriptional regulator, LysR family	amber nonsense codon	157
VF_A0466	VFA0466	N-acetylglucosaminyltransferase	-1 frameshift	177

There is little information about the remaining four pseudogenes, except for *umuC*. The transcriptional organization between the genes encoding the DNA polymerase V subunits *umuD *and *umuC *is conserved between ES114 and MJ11 (Additional file 5). However, *umuC *has uniquely degenerated in ES114, with both a nonsense codon and a 5-bp repeat expansion following the nonsense codon. DNA polymerase V is responsible for error-prone translesion synthesis (e.g., following UV-irradiation), which allows DNA synthesis to proceed despite a high rate of error incorporation [32], yet there are organisms, including *V. cholerae *El Tor, that apparently do not encode these functions [33,34]. Whether the situation in ES114 represents an evolutionary transition state, or instead this arrangement (*umuD*^+^*umuC*^-^) has relevant functional implications remains to be determined.

### Annotation of previously uncalled protein-coding genes, regulatory RNAs, and operon leader peptides

Because examination of the intergenic region corrected by target no. 172 revealed a likely protein-coding gene, we asked whether there were other genes present within the ES114 sequence that were previously unannotated. Additionally, regulatory RNA genes had not been previously annotated in the *V. fischeri *genome, yet they are known to play important roles in *V. fischeri *and other diverse bacteria [35,36]. Therefore, we undertook an effort to systematically identify ORFs and regulatory RNA genes that had not been called in the published version 1.0 sequence.

To accomplish this search we took advantage of the annotations present in the J. Craig Venter Institute's Comprehensive Microbial Resource (JCVI CMR), which include *ab initio *gene-calls that can differ from those in the deposited GenBank flatfiles [37]. We examined the list of approximately 150 novel genes identified in the CMR. We excluded candidates that were unlikely to be biologically significant – generally, short ORFs that were encoded on the opposite strand against much larger ORFs – and were left with 53 likely novel ORFs (Additional file 4, Basis code "A"). We also took advantage of the presence of the closely-related MJ11 strain as a source for novel gene annotations. Of the MJ11 proteins that did not have an ortholog in ES114, we examined those in which a TBLASTN query of MJ11 proteins against the ES114 genome yielded a percent amino acid identity value of at least 85%. We excluded candidates in which there was low biological basis for the assignment (as described above), or in which the open reading frame was not conserved in ES114. There were 72 novel ES114 ORFs assigned by comparison with MJ11 (Additional file 4, Basis "B"). 30 ORFs were called by both methods (CMR and MJ11 conservation), whereas 65 genes were called by only one method, for a total of 95 uncalled chromosomal protein-coding genes that we predict to be uncalled in ES114 (Table 3, Additional file 4).

**Table 3:** Summary of 113 new gene features in ES114 version 2.0.

	Regulatory RNAs	Operon leader peptides	Protein-coding genes	**TOTAL**
Chromosome I	9 (9)^*a*^	6 (6)	73 (13)	**88 (28)**
Chromosome II	1 (1)	0 (0)	22 (3)	**23 (4)**
**CHROMOSOMES TOTAL**	**10 (10)**	**6 (6)**	**95 (16)**	**111 (32)**
				
Plasmid pES100	0 (0)	0 (0)	2 (2)^*b*^	**2 (2)**

Numbers in parentheses indicate subset of features that have an annotation other than "hypothetical."

^*a *^Includes *csrB1 *and *csrB2 *[36].

^*b *^Includes two genes predicted from [61].

In all of these cases, no sequence was changed in the genomic model, but annotations imposed on the sequence were added. Included in the list of new genes predicted from both approaches is biotin synthase (*bioB*). Because ES114 grows on minimal medium lacking biotin [38], BioB is likely expressed by the organism. Another example of a gene predicted from both approaches is the oxaloacetate decarboxylase gamma subunit (*oadC*), which is predicted to be encoded in an operon with the already-predicted alpha and beta subunits (new operon prediction of *oadCAB*).

In addition to genes that were identified from both the MJ11-comparative and JCVI CMR approaches, we believe that genes identified by only one of the approaches are still worthy of inclusion, subject to the filters imposed above. Genes that were identified by comparison with MJ11 have the support that the open reading frame is conserved in at least these two strains. A similar measure has been used to call genes in *Saccharomyces cerevisiae *for genome inclusion [39]. Genes that were identified solely from the JCVI CMR annotation include a number of regions that are unique to ES114, such as a prophage that is present in ES114 and absent in MJ11 (Additional file 4, new loci VF_2640 through VF_2649), and therefore would not be expected to be called by comparison with MJ11. The coding density of the prophage was markedly increased due to the addition of the novel gene annotations, consistent with phage genome organization and supporting the assignments predicted by the CMR. It is clear that the consolidated results from both methods, though partially overlapping, identify a significant number of novel, bona fide gene annotations in ES114.

We added regulatory RNA genes to the annotation as identified from multiple sources (Table 3, Additional file 4). Prediction of CsrB regulatory RNAs has been pioneered using *V. fischeri *[36], and additional regulatory RNAs were added based on motifs found in the RFAM database [40]. These methods identified a total of 10 regulatory RNAs and 1 operon leader peptide. Although operon leader peptide predictions are not typically found in databases, we speculated that additional such genes were present in ES114 and, using the Ecocyc database [41] as a guide, we manually searched for operon leader peptides in ES114 and identified five additional high-confidence members in the genome (Additional file 4, Basis "E").

In total, we called 95 new protein-coding genes, 8 regulatory RNAs, and 6 operon leader peptides, and we incorporated 2 protein-coding genes and 2 regulatory RNAs that were published previously, for a total of 113 new annotations incorporated into ES114 version 2.0 (Table 3, Additional file 4).

### Comprehensive annotation update

In the process of correcting sequence errors and adding missing annotations, we additionally took the opportunity to update the annotations of the genes in the ES114 genome and to establish a framework for future genomic and genetic studies in *V. fischeri*. To update the product annotations of *V. fischeri*, we assembled a database of *V. fischeri *genetic and genomic analyses from the the PubMed database [42]. Our initial curated *V. fischeri *list included 545 unique gene-publication associations from 60 publications, encompassing 339 distinct genes represented in strain ES114. This list served as the core of the reannotation effort, which further gave us the opportunity to update a number of genes whose functions have been discovered since the initial genome publication.

For all genes in ES114, we additionally compared protein annotations from multiple sources: (1) Orthologous protein annotations in the recently reannotated *Escherichia coli *K-12 MG1655 sequence, and updates made subsequent to sequence publication through the ASAP and Ecocyc databases [41,43]; (2) Orthologous protein annotations in *V. cholerae *[34]; (3) the JCVI CMR [37]; and (4) UniprotKB [44]. These comparisons allowed us to update the annotations and to make the annotations more consistent with current practice and NCBI guidelines.

We found the annotations of *E. coli *– though most distant phylogenetically – to be the most valuable empirically. The timeliness of the update and the availability of curated, referenced descriptions in the Ecocyc entries allowed us to improve a number of entries that appeared to lag behind the other data sources. As one example, we point to the case of *yihY *(VF_0100, ortholog of *E. coli *locus tag b3886). Previously annotated as encoding the ribonuclease BN [45], this annotation has been propagated through numerous sources, including most of the *Vibrionaceae *genomes. A subsequent report identified the *E. coli rbn/elaC *gene (locus tag b2268) as the gene that encodes RNase BN, and the most recent genome annotation for b3886 has been updated as *yihY*, "predicted inner membrane protein" [46]. We compared data from the sources described above, as well as the literature described, and captured this update by calling VF_0100 as *yihY *with a product of "predicted inner membrane protein". In fact, *V. fischeri*, like most sequenced *Vibrio *spp., does not contain an *rbn *ortholog, and therefore having any product labeled as "ribonuclease BN" would have been misleading from the perspective of predicting genome capabilities. We note that the old annotation persists in major databases [31,47-49] and in most of the *Vibrionaceae *genomes available at the time of data submission. This example highlights the value and relevance of the *E. coli *K-12 update to this and related annotation projects, as well as our ability to capture the latest information about genes encoded in the ES114 genome.

Fine-scale annotation changes, such as those shown in Figure 1F, are detailed both in that figure and in the Methods. We also wish to highlight the updated entry for *prfB *(noted in Table 3), the peptide chain release factor RF-2. The programmed frameshift in *prfB *is not called correctly by machine-call algorithms, and this gene is improperly entered in the GenBank flatfiles of all of the previously submitted *Vibrio *spp. genomes.

With the blossoming number of sequencing projects, utilization of locus tags (e.g., VF_0001) as identifiers for both genes and their products has become commonplace as the increase in genomic characterization has outpaced genetic and biochemical characterization of gene products. Nonetheless, biological analysis in a genomic context depends on understanding gene function, and proper nomenclature has been adopted in a number of species to facilitate meaningful communication about genes and their products. In fact, we (and others) repeatedly refer to genes by their identifiers and, without tracking in a database, this practice can lead to incorrect conclusions [50]. Therefore, whereas the previous ES114 version did not contain 3–5 character "gene" identifiers, we added those for approximately 1,995 genes in which the identity of the gene could be identified or inferred from published work in *V. fischeri*, or by orthologous genes in other organisms. Due to the availability of well-curated database resources, most of the names were derived from their orthologs in *E. coli *MG1655 [41,43,51].

We demanded that unique gene identifiers be a minimum of three lowercase letters (e.g., *fnr*), with an optional uppercase letter (e.g., *dnaA*), and/or an optional numeral (e.g., *nagA2*), for a maximum of five characters total. Such numeric suffixes were assigned to distinguish among members of paralogous families or genes of related function. For approximately half of the genes, no gene identifier could be assigned at this time.

### *V. fischeri *transcription machinery

Because three RNA polymerase subunit genes were affected by the resequencing (*rpoB*, *rpoC*, *rpoH*), we took a genomic inventory of the corrected ES114 transcriptional apparatus in a manner that was not possible prior to the targeted resequencing. The subunits identified in the genome are listed in Table 4 and include the core subunits α, β, β', and ω. Classification of the eleven identified sigma factors is described below and was performed by the scheme outlined in Gruber & Gross [52].

**Table 4:** ES114 genes encoding transcriptional machinery.

**Locus_tag**	**Gene**	**Product**	**Notes**
***RNA polymerase core***			
VF_0262	*rpoA*	α subunit	
VF_2414	*rpoB*	β subunit	
VF_2412	*rpoC*	β' subunit	
VF_0105	*rpoZ*	ω subunit	
			
***Sigma subunits (11 predicted)***			
VF_2254	*rpoD*	σ^D^/σ^70^	Group 1: σ^70^-type
VF_2067	*rpoS*	σ^S^	Group 2: σ^70^-type, σ^38^-subtype
VF_A1015	*rpoQ*	σ^Q^	Group 2: σ^70^-type, σ^38^-subtype
VF_2450	*rpoH*	σ^H^	Group 3: σ^70^-type, σ^32^-subtype
VF_1834	*fliA*	σ^F^	Group 3: σ^70^-type, σ^28^-subtype
VF_2093	*rpoE*	σ^E^	Group 4: σ^70^-type, σ^24^-subtype
VF_0972	*rpoE2*	σ^E2^	Group 4: σ^70^-type, σ^24^-subtype
VF_A0820	*rpoE3*	σ^E3^	Group 4: σ^70^-type, σ^24^-subtype
VF_A0766	*rpoE4*	σ^E4^	Group 4: σ^70^-type, σ^24^-subtype
VF_2498	*rpoE5*	σ^E5^	Group 4: σ^70^-type, σ^24^-subtype
VF_0387	*rpoN*	σ^N^	σ^54^-type

Group 1 sigma factors include regions 1.1, 1.2, 2, 3, and 4. This category includes only σ^70 ^in ES114. Group 2 sigma factors (regions 1.2, 2, 3, 4) include the closely-related σ^S ^subunits; as mentioned above, *V. fischeri *curiously contains two of these sigma subunits. In addition to a clear ortholog of *rpoS *(VF_2067), the gene encoding the stationary-phase sigma subunit (σ^S^), ES114 also contains a gene that is expected to encode a σ^S^-like subunit (VF_A1015). Transcript levels of this second σ^S^-family subunit increase upon C8-homoserine-lactone (AinS-dependent) quorum-sensing [18], so we have called the product σ^Q ^(encoded by *rpoQ*) to designate this as a quorum-responsive sigma factor and to distinguish it from the σ^S ^paralog. Group 3 sigma factors (regions 2, 3, 4) include the specialized sigma factors σ^H ^and σ^F^. Group 4 sigma factors (regions 2 and 4 only), also called ECF sigma factors because they perform an extra cytoplasmic function in responding to envelope stresses, are the most divergent. ES114 contains five of these subunits, with the corresponding genes named *rpoE*, *rpoE2*, *rpoE3*, *rpoE4*, and *rpoE5*. Although these have not yet been studied in *V. fischeri*, their genomic context suggest function in some cases. Unlike the other Group 4 sigma factors, the product of the gene called *rpoE *is a close homolog of *E. coli *σ^E ^(79% identical, 91% similar) and is organized transcriptionally with regulatory genes homologous to its *E. coli *counterparts (*rseA, rseB, rseC*). We assigned this subunit σ^E^, and this subunit may respond to outer membrane protein misfolding in a similar manner as in *E. coli *[53]. *rpoE4 *is predicted to be transcribed in an operon with *chrR*, which likely encodes an anti-σ^E4 ^factor based on homology with the reactive oxygen-sensing σ^E^/ChrR system of *Rhodobacter sphaeroides *[54]. Additionally, the two genes flanking *rpoE5*, though their functions are unknown, share a similar phylogenetic distribution as *rpoE5*. Because Group 4 sigma factors are typically cotranscribed with cognate anti-sigma factors [52], evolutionary co-inheritance of this three-gene cassette supports a role for the surrounding genes in regulating the levels and activation of σ^E5^.

## Discussion

We initiated this study to clarify the status of a number of suspect ORFs in a completed genome sequence of an organism that is of value for studies on bacterial communication and host interaction. Of the 4.3 Mbp of chromosomal DNA in the original, version 1.0 release, 0.2% of the sequence was in error, mostly due to fourteen regions (ranging from 318 bp to 1264 bp) in which unique DNA was incorporated in tandem in the version 1.0 release. A total of 174 individual sites were corrected – by insertion, deletion (large or small), or substitution – leading to changes in 137 protein-coding loci of the version 1.0 release (3.6% of total ORFs), and one newly-annotated ORF. Salzberg and Yorke [55] describe "compressions" that can occur in eukaryotic genome assemblies when errors compress multiple, repeated sequences and exclude intervening unique regions. The type of error that we have detected is an "expansion" in that sequence is illegitimately repeated and the resulting region has been expanded.

The initial shotgun sequencing and assembly for ES114 were conducted circa 2001. However, sequence quality varies by project and center/investigator, and the problems encountered here are neither unique to (nor necessarily as prevalent among) older genomes. For example, as we identified resequencing targets in ES114, we additionally targeted regions in the draft *V. fischeri *MJ11 sequence as part of our ongoing effort to close an accurate MJ11 sequence. Of fifteen loci targeted for resequencing, nine were found to contain point mutations that led to frameshifts (data not shown). These data will be incorporated into the complete MJ11 sequence. Additionally, a systematic analysis to identify possible gene errors of the type we describe here identified interrupted genes in other complete genomes from Gram-negative γ-proteobacteria, and in some of those cases the interrupted genes are orthologs of essential genes in *E. coli *[8,26]. In cases where essential genes may be predicted from related organisms, we propose that measuring the proportion of such genes that are interrupted could serve as a measure of quality assessment for newly-assembled genomes. In the case of *V. fischeri *ES114, identifying interrupted essential genes was a significant clue that the sequence model required refinement, and those genes represent 12 of the 74 (16%) loci corrected (Table 1). The comparative approach that we describe was necessary to identify the additional affected loci.

In addition to the *E. coli *and *M. smegmatis *resequencing/reannotation projects discussed earlier, a comparative-based reannotation project has been reported in *S. cerevisiae *[39]. Our approaches and results are similar to the yeast study in that both relied heavily on comparison with a single additional genome as a basis for gene discovery and clarification. In addition to a draft genomic sequence of a closely-related sequence, we relied solely on publicly-available data to compile our annotations and complete this update. Thus, the methods described here are generally applicable in any case in which there is updated GenBank data for closely-related organisms.

The timeliness of any genome update is necessarily transient, and therefore it is of community interest to optimize the data quality and usability of annotation systems. To track future sequence and annotation changes in *V. fischeri*, we have established a web site that will assist in coordinating *V. fischeri *annotation resources and genome projects into the future [56].

Because GenBank functions mainly as a deposition library – and not as a dynamic annotation interface – development of such an interface would enhance the ability to keep genome data current. If such a resource were to be developed, annotations could be propagated in a manner that can be intelligently curated by individual genome owners, and could be managed through a user-friendly interface. The development of Bioinformatics Resource Centers (BRCs) (e.g., [57]) has advanced the annotation pipelines for pathogenic microorganisms, but this system is insulated from many of the investigators who work on the vast majority of organisms represented in the database, and requires consistent deposition in GenBank for there to be a "paper trail" that can be accessed by outside investigators. Additionally, establishing a distinction between human-pathogenic and human-non-pathogenic organisms in genome annotation is artificial when the organisms overlap phylogenetically and significant work is underway to characterize similar (often the same) gene products and pathways in related organisms. It would be beneficial to the prokaryotic genome community to find a way to integrate genomic data into a dynamic resource without regard to the human-pathogenic phenotype of the organism.

## Conclusion

As DNA sequencing technology accelerates, it is critical to ensure that high-fidelity sequence assemblies are obtained, and that large-scale errors be readily detected. By using publicly-available resources and the sequence (even the draft genomic sequence) of a close relative, errors that lead to miscalling of ORF boundaries in one strain relative to that of another may be identified for regions syntenous between the strains. Such analyses can identify regions that were assembled incorrectly, or even point mutations that were miscalled in one strain. This analysis in *V. fischeri *ES114 identified over 10 kb of sequence that required adjustment, including 14 regions requiring deletion from the annotation, and overall errors affecting 138 ORFs across 74 loci.

It is similarly important to maintain accurate and updated annotations for genes in sequenced genomes. Although some extensively-studied model organisms have systematic programs for their annotation, organisms with sparser genomic resources – and often, fortunately for this purpose, fewer data generated from direct studies in that organism – can benefit from a streamlined reannotation pipeline using recently updated annotations from publicly-available databases. By applying such an approach, we updated the complete annotation of *V. fischeri *ES114 and included regulatory RNAs and operon leader peptides, important regulatory features that are commonly missed by automated gene calling. Although community-based updates are desirable when they can be accomplished, our individual approach is generally applicable across microbial genomics and demonstrates a straightforward way to achieve a high-yield update with resources that are common in hundreds of laboratories.

## Methods

### Identification of suspect regions by reciprocal BLASTP analysis

We compared the predicted proteomes from both ES114 chromosomes against the chromosomal contigs of MJ11. Reciprocal exhaustive BLASTP [42] searches were performed with an expect cutoff of 10. Results were filtered to demand that the query length and subject length each be a minimum of 60% of their respective total lengths. Among the remaining results for each query protein, best-hits were scored by percent amino acid identity, and additional results were included for analysis if they scored at least 70% of the maximum score for that query. ES114-MJ11 protein pairs included on reciprocal lists were candidate orthologs, and for the <200 pairs in which there was a duplicate of query or subject protein, manual assignment of orthology was curated using the parameters of percent amino acid identity, percent of each protein aligned, and the local genomic context (synteny) of the two proteins, which was possible to determine in most cases even though MJ11 was in draft format. Curation of this list resolved many of the duplicates satisfactorily; the remaining duplicates are the subject of this study. We note that the effect of this analysis is similar to that employed by Perrodou et al. [8]. The plasmid proteins were dissimilar between the two genomes and in the absence of a strong reference sequence were not analyzed extensively for putative sequence errors.

### Sequence clarification

*V. fischeri *strain ES114 (isolate MJM1100) was used for all of the sequence analysis except as noted. This isolate is a first-generation descendent of the ES114 which served as the source of genomic DNA for the original ES114 sequencing project. We know of no phenotypic or molecular distinction between the two strains. Genomic DNA was prepared using the MasterPure Complete DNA Purification Kit (Epicentre, Madison, WI).

PCR amplification was conducted using Platinum Taq DNA Polymerase High-Fidelity (Invitrogen, Carlsbad, CA). Fifty-μl reactions contained: 250 ng ES114 genomic DNA, 1× reaction buffer, 0.2 mM of each dNTP, 2 mM MgSO_4_, 0.25 μM of each primer, and 1 U DNA Polymerase. Thermal cycling was conducted in a PTC-200 thermal cycler (MJ Research, Watertown, MA): 95°C for 2:00; then 30 cycles of 95°C for 0:30, 55°C for 0:30, 68°C for 0:30–1:00 per kb amplified; then 68°C for 5:00. Most primer pairs (full list in Additional file 1) amplified products in the range of 500–1000 bp and, thus, we used an extension time of approximately 0:30. Where the product was greater than 1 kb, at least three independent PCR reactions were combined for sequencing to minimize the effect of PCR error.

Sequencing was performed at both the University of Washington High-Throughput Genomics Unit (Seattle, WA) and the University of Wisconsin Biotechnology Center DNA Sequencing Facility (Madison, WI). Analysis of the sequence of the regions surrounding each suspect area revealed patterns of polymorphisms that were ES114-specific – that is, outside of the region of suspected sequencing error (which often locally resembled MJ11), the remainder of the resequenced product was distinct from MJ11, and identical to the published ES114 sequence. This observation supported the notion that there were discrete errors in the previously-published ES114 sequence, that we were able to isolate and correct the problem sequence, and that the problems described were isolated within clear margins of discrepancy. A detailed inventory of the sequence changes to create ES114 version 2.0 may be found in Additional file 2.

### Addition, removal, and annotation of protein-coding genes

Principal sources of additional ES114 gene annotations since the initial publication of the genome sequence include description of the *syp *polysaccharide cluster [58], the *mif *diguanylate cyclase genes [59], several two-component systems [19], and an inventory of predicted flagellar and chemotaxis genes [60]. In addition, Dunn et al. [61] annotated two new genes on the ES114 plasmid pES100 as VFB38.5 and VFB39.5; these locus tags were adjusted for consistency with NCBI guidelines to VF_B0056 and VF_B0057, respectively.

In this study, novel genes were added from a subset of the genes listed in the ES114-specific dataset at the JCVI CMR [37] as described in the text. Genes in MJ11 that had unannotated orthologs in ES114 were identified by selecting MJ11 genes that failed to identify an ortholog as described above, and performing TBLASTN [24] queries against the ES114 genome. High-scoring results (>85% amino acid identity) in which the open reading frame was conserved between the two strains were designated as novel genes in ES114.

Annotation updates to chromosomal protein-coding genes were curated from the JCVI CMR and from Uniprot-KB. We also considered gene and protein annotations from orthologs in *Escherichia coli *K-12 MG1655 (GenBank accession no. U00096.2) [51] – including updates made subsequent to sequence publication through the ASAP [43] and Ecocyc [41] databases – and orthologous protein annotations in *V. cholerae *N16961 (GenBank accession nos. AE003852.1, AE003853.1) [34].

Fine-scale annotation changes included refinement of gene boundaries in 16S rRNA genes as shown in Figure 1F. Eleven small coding sequences (VF0334, VF0335, VF0567, VF2127, VF2160, VF2429, VF2430, VF2431, VF2530, VF2531, VF2532) that overlapped 23S rRNA genes were deaccessioned because they were unlikely to represent true ORFs. Other genes that were deaccessioned are listed where appropriate in the corresponding tables.

### Addition and annotation of genes encoding RNAs

The *csrB1 *and *csrB2 *RNA gene annotations were designated by Kulkarni et al. [36], and the *qrr1 *annotation was identified by homology with the gene described in Lenz et al. [62]. Additional regulatory RNA genes were identified using the RFAM database, with subsequent information and alignments from multiple primary and secondary sources. Because experimental validation of most prokaryotic noncoding RNAs has occurred in *E. coli *K-12, we relied on that organism's sequences to predict regulatory RNA gene boundaries in *V. fischeri*. For this update we did not include riboswitches and other *cis*-regulatory elements, except those that are transcribed as separate genes (see next section).

### Annotation of operon leader peptides

The histidine leader-peptide gene *hisL *was predicted based on the annotated feature in RFAM; others were found by sequence gazing, guided by known operon leader peptides in *E. coli *K-12, as annotated in the Ecocyc database [41]. Peptides with appropriate amino acid density (or thymine density, in the case of *pyrL*) in regions comparable to their homologs in *E. coli*, were annotated as operon leader peptides.

### Sequence information and versioning

The updated NCBI Genomes database files for *V. fischeri *ES114 are [GenBank:CP000020, GenBank:CP000021, GenBank:CP000022]. The files were accepted by NCBI on 10/03/2007 (GenBank x.2 version of each), and references to data sources are accurate as of that date. We have assigned those files as release version 2.0. The individual resequenced fragments were deposited in the NCBI GSS database, under the accession numbers listed in Additional file 3.

The *V. fischeri *MJ11 draft genome package has been deposited as [GenBank:NZ_ABIH00000000]. Updated assembly and correction information is available at the *V. fischeri *Genomics Site [56].

## Supplementary Material

Additional file 1 Table listing oligonucleotide primers. The PCR and sequencing primers used to analyze resequencing targets. Click here for file

Additional file 5 Figure of *umuDC *degeneration in ES114. Alignment of *umuDC *in strains ES114 and MJ11. Click here for file

Additional file 4 Table of novel gene features. Detailed ES114 gene annotations added in version 2.0. Click here for file

Additional file 2 Table of version 2.0 sequence changes. Detailed base-by-base descriptions of sequence changes from ES114 release version 1.0 to 2.0. Click here for file

Additional file 3 Table of fragment GenBank accession numbers. The GenBank accession numbers for the resequenced fragments in ES114. Click here for file
