# Supplementary figures and images for: Comparative genomics-based investigation of resequencing targets in Vibrio fischeri: Focus on point miscalls and artefactual expansions
Source: BMC Genomics. 2008 Mar 25;9:138. doi: 10.1186/1471-2164-9-138 (PMC2330054; doi:10.1186/1471-2164-9-138)

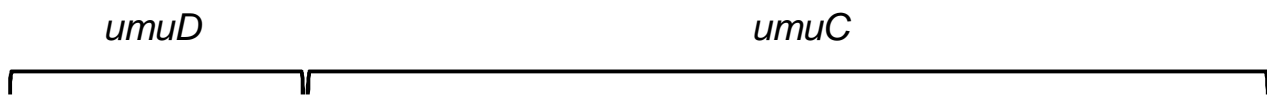

**ES114**

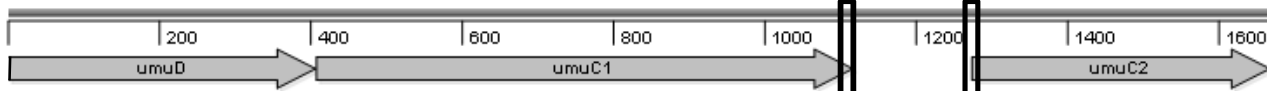

**MJ11**

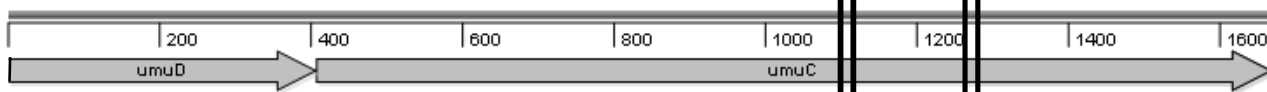

**ES114**  
**MJ11**

...TAG...  
...TGG...

...CTT TGCTT TGCTT TGCTT CAAA ...  
...CTT TGCTT TGCTT ----- CAAA ...

Supplement: Additional file 5 — Figure of umuDC degeneration in ES114. Alignment of umuDC in strains ES114 and MJ11. [file 1471-2164-9-138-S5.pdf]
